# Impact of district-level comprehensive tobacco control on smoking among adolescent boys in Indonesia: a synthetic control study (2013–2023)

**DOI:** 10.1016/j.lansea.2026.100798

**Published:** 2026-06-11

**Authors:** Dian Kusuma, Hario Megatsari

**Affiliations:** a Department of Public Health and Epidemiology, College of Medicine and Health Sciences, Khalifa University of Science and Technology, Abu Dhabi, United Arab Emirates; b Center for Biotechnology, Khalifa University of Science and Technology, Abu Dhabi, United Arab Emirates; c Department of Health Education and Behavioral Science, Faculty of Public Health, Universitas Airlangga, Surabaya, Indonesia

**Keywords:** Tobacco control, Smoking, Adolescents, Synthetic control, Indonesia, Health disparities, Policy evaluation, District-level analysis

## Abstract

**Background:**

Indonesia has one of the highest smoking prevalences globally, particularly among boys and men, yet national tobacco control implementation remains limited. We aimed to evaluate the impact of comprehensive district-level tobacco control measures on smoking prevalence among male adolescents in a decentralized policy environment.

**Methods:**

We applied a quasi-experimental synthetic control approach using panel data from 497 Indonesian districts between 2013 and 2023. Bogor City, which intensified local tobacco control measures from 2014 onward, served as the treated unit. Smoking prevalence data were drawn from nationally representative Riskesdas surveys (2013, 2018, 2023). Pre-intervention covariates measured in 2013—including socioeconomic, educational, and health service indicators—were used to construct synthetic controls. Robustness was assessed through sensitivity analyses and placebo tests. The primary outcomes were ever and current smoking among male adolescents; adult men were examined as a secondary outcome.

**Findings:**

Compared to synthetic controls, Bogor City experienced reductions in smoking prevalence among male adolescents: a 14.7 percentage point reduction in current smoking (43.4% relative reduction) and a 12.3 percentage point reduction in ever smoking (34.5% relative reduction). These gaps widened over time, suggesting sustained policy impact. Among adult men, the effect was more modest: ever smoking declined by 3.2 percentage points (3.9% relative reduction), whereas current smoking prevalence remained 11.3 percentage points higher than the synthetic control (19.5% relative difference), consistent with limited responsiveness among established smokers.

**Interpretation:**

District-level comprehensive tobacco control was associated with reductions in smoking among male adolescents in Indonesia, despite weak national enforcement. These findings underscore the potential of subnational action in preventing youth smoking and support the need for complementary national measures to strengthen tobacco control.

**Funding:**

Khalifa University of Science and Technology.

**Research in context**

**Evidence before this study**

We conducted a structured search of Google Scholar for articles published from Jan 1, 2000, to Dec 31, 2025, using combinations of the terms “tobacco control”, “smoke-free law”, “advertising ban”, “TAPS”, “synthetic control”, “quasi-experimental”, “subnational”, “district-level”, “Indonesia”, “Southeast Asia”, and “youth smoking”. Extensive evidence shows that comprehensive national tobacco control policies—particularly smoke-free laws, taxation, and advertising bans—reduce smoking prevalence and improve health outcomes. Synthetic control methods have been used to evaluate national tobacco reforms in several settings. However, evidence on the effectiveness of subnational tobacco control in decentralized systems with weak national implementation remains limited. In Indonesia, prior studies have largely been descriptive or cross-sectional, focusing on policy adoption, compliance, or smoking patterns. We found no rigorous counterfactual evaluation of district-level tobacco control implementation in Indonesia.

**Added value of this study**

This study provides a quasi-experimental evaluation of district-level comprehensive tobacco control in Indonesia using a synthetic control framework. By leveraging nationally representative district-level data over a decade, it moves beyond descriptive analyses to estimate counterfactual smoking trends. The findings demonstrate that strong subnational action can meaningfully reduce youth smoking in a decentralized and weakly regulated national context. The study also highlights differences between youth initiation and adult cessation responses, contributing to understanding how distinct tobacco control instruments operate across population groups.

**Implications of all the available evidence**

Together with existing evidence, our findings suggest that subnational tobacco control policies can generate measurable public health gains, particularly in preventing youth smoking initiation, even where national enforcement is limited. However, sustained reductions in adult smoking likely require complementary national measures, including substantial excise taxation and accessible cessation support. In decentralized systems, empowering local governments to implement comprehensive tobacco control can serve as a critical strategy while broader national reforms are strengthened. Future research integrating quantitative and qualitative approaches is needed to better understand implementation dynamics and gender-specific impacts of subnational tobacco control policies.

## Introduction

Tobacco use continues to be a major cause of preventable illness and death globally. Each year, it is responsible for over 7 million deaths, including approximately 1.6 million non-smokers who die due to exposure to second-hand smoke.^1^ The Global Burden of Disease (GBD) Study 2021 ranked smoking as the second leading risk factor for attributable deaths and disability globally.^2^ The burden is particularly pronounced in low- and middle-income countries (LMICs), where over 80% of the world's 1.3 billion tobacco users reside.^1^^,^^3^

Indonesia has one of the highest smoking prevalences globally, particularly among boys and men. According to the 2023 Indonesia Health Survey (SKI), 9.7% of boys aged 10–18 had ever smoked, and 8.6% were current smokers. Among adult men, current smoking prevalence stood at 62.7%—one of the highest globally.^4^ In contrast, smoking prevalence among women and girls remains substantially lower across age groups, resulting in limited variability and statistical instability at the district level.^4^ Consequently, this study focuses on male adolescents and adult men, among whom smoking prevalence is sufficiently high to permit robust subnational evaluation. Despite the significant health burden caused by tobacco use, Indonesia remains one of the few countries that has not ratified the WHO Framework Convention on Tobacco Control (FCTC)^5^ and has yet to fully implement key measures such as a comprehensive national tobacco control law, nationwide outdoor advertising bans, or substantial tobacco taxation.^6^, ^7^, ^8^ As a result, tobacco control efforts in Indonesia are largely fragmented and decentralized, with considerable variation in implementation across districts and a heavy reliance on local government leadership to drive policy action.^9^, ^10^, ^11^

Bogor City, located in West Java Province, has emerged as a national pioneer in comprehensive tobacco control. It joined the Bloomberg Initiative in 2008 and partnered with the International Union Against Tuberculosis and Lung Disease to become a 100% smoke-free city. In 2009, Bogor passed Indonesia's first comprehensive smoke-free law, banning smoking in all indoor public spaces, workplaces, public transport, and all forms of tobacco advertising and promotion, including point-of-sale displays.^12^, ^13^, ^14^ To ensure effective enforcement, the city issued a mayoral decree in 2010 outlining detailed implementation steps, including random inspections and mobile courts. In 2014, Bogor expanded its efforts through a mayoral regulation that banned all forms of outdoor tobacco advertising, promotion, and sponsorship, followed by a point-of-sale display ban in 2017, which achieved over 90% compliance. Strong political leadership, multi-sectoral coordination, civil society engagement, and active enforcement mechanisms have enabled Bogor to implement one of the most advanced local tobacco control programs in Indonesia—preceding and exceeding national regulations.^12^, ^13^, ^14^

Previous studies have evaluated national-level tobacco control policies in countries with strong programs, such as Thailand and South Africa, demonstrating significant reductions in smoking and improved child health outcomes. In South Africa, tax-led tobacco control measures between 1994 and 2004 reduced per capita cigarette consumption by 36%.^15^ In Thailand, comprehensive smoke-free legislation introduced in 2010 led to annual reductions of 2.9% in neonatal and 2.8% in infant mortality.^16^ More broadly, Radó and colleagues found that such legislation across 106 middle-income countries reduced neonatal and infant mortality by 1.63% and 1.33% annually, underscoring the substantial health gains from robust tobacco control.^17^

However, evidence on the effectiveness of subnational interventions—particularly in countries with weak national tobacco control like Indonesia—is limited. No rigorous evaluation exists for Indonesia, where most studies are descriptive or cross-sectional and lack counterfactual analysis.^18^, ^19^, ^20^, ^21^ Moreover, district-level differences in socioeconomic and health infrastructure complicate simple before-and-after comparisons.

This study addresses these gaps by applying the synthetic control method to estimate the causal effect of district-level tobacco control implementation on smoking outcomes, leveraging the case of Bogor City—a local leader in comprehensive tobacco control—to assess the health impact of strong local action within a weak national policy environment. By rigorously estimating counterfactual trends, it contributes new evidence on the real-world effectiveness of local tobacco control in Indonesia, offering important insights for scaling similar policies at the national level.^15^, ^16^, ^17^

## Methods

This study employed a quasi-experimental synthetic control method to estimate the impact of district-level comprehensive tobacco control policies in Indonesia. Bogor City served as the treated unit following its 2014 ban on tobacco advertising, promotion, and sponsorship (TAPS). Using panel data from 497 Indonesian districts spanning 2013–2023, we constructed a synthetic control to estimate how smoking prevalence in Bogor City would have evolved in the absence of the intervention.

Smoking prevalence data were obtained from the Indonesia Health Surveys (Riskesdas or SKI) for 2013, 2018, and 2023, which are nationally representative and stratified at the district level. Prevalence was measured separately for male adolescents (ages 10–18 years) and adult men (ages 19+), for both current and ever smokers. District-level covariates measured in 2013 were sourced from the World Bank's Database for Policy and Economic Research (DAPOER). These included the human development index, poverty rate, adult literacy, school enrollment (primary, junior secondary, senior secondary), and the ratios of primary health centers (Puskesmas) and hospitals to population.

We applied a two-step exclusion process to define the donor pool. First, we excluded 10 districts known to have implemented strong tobacco control measures during the study period (six districts within Jakarta Province, as well as Depok City, Klungkung Regency, Padang Panjang City, and Kulon Progo Regency) to avoid contamination of the counterfactual. Second, to improve structural comparability and reduce extrapolation from markedly dissimilar districts, we restricted the donor pool to districts in the top two quintiles of the human development index (HDI) in 2023, reflecting structural socioeconomic comparability with Bogor City.^15^^,^^16^

We excluded the 10 tobacco-control districts because their policy adoption was staggered and heterogeneous in timing, scope, and enforcement, making it difficult to define a single, uniform treatment exposure; pooling them with Bogor City into a composite treated unit could compromise internal validity.^22^ In addition, including these districts in the donor pool could introduce contamination by incorporating partially treated units into the counterfactual, thereby biasing effect estimates toward the null. We therefore focused on Bogor City as a sentinel case of early, comprehensive tobacco control implementation. The intervention was defined as the 2014 implementation of Bogor City's outdoor TAPS ban; however, because smoking prevalence was observed in 2013, 2018, and 2023, 2013 served as the pre-intervention baseline and 2018 was the first post-intervention observation (and was specified as the intervention period in the synthetic control model).

The primary outcomes were the prevalence of ever and current smoking among male adolescents. Secondary outcomes included ever and current smoking among adult men. These outcomes were assessed using data from three nationally representative health surveys—Riskesdas 2013, 2018, and 2023—allowing for both pre- and post-intervention comparisons over time.^4^^,^^19^ The key independent variable was a binary treatment indicator, with Bogor City coded as treated from 2014. Synthetic controls were constructed using Stata's -synth- command, which assigns weights to donor districts to best match Bogor's pre-intervention characteristics and smoking trends.^15^, ^16^, ^17^

### Statistical analysis

We compared smoking prevalence trends between Bogor City and its synthetic control and examined post-intervention gaps (treated minus synthetic) over time. Absolute and relative differences in prevalence were calculated for 2023.

To assess robustness, we conducted sensitivity analyses by re-estimating the synthetic control model using the full donor pool without exclusions, to evaluate whether results were sensitive to donor pool restrictions.

We further assessed statistical significance using placebo (permutation) tests following established synthetic control practice. In this approach, the intervention was iteratively reassigned to each donor district, treating each as if it were exposed to the policy in 2014, with synthetic controls re-estimated using the remaining districts. This procedure generates a distribution of post-intervention gaps under the null hypothesis of no treatment effect.

To ensure meaningful comparison, inference was restricted to placebo districts with adequate pre-intervention fit. Pre-intervention fit was assessed using the root mean squared prediction error (RMSPE) between observed and synthetic outcomes in 2013, the only available pre-intervention time point. Placebo districts were considered to have “good fit” if their pre-intervention RMSPE was less than or equal to that of Bogor City, which was retained in all comparisons.

Pseudo p-values for 2023 were calculated as the proportion of placebo districts with good pre-intervention fit whose absolute post-intervention gap was greater than or equal to that observed for Bogor City. This permutation-based approach provides a non-parametric assessment of the likelihood that the observed effects could arise in the absence of the intervention, consistent with established practice in synthetic control analyses.^15^, ^16^, ^17^

In constructing the synthetic control, Bogor City was matched to a weighted combination of donor districts based on pre-intervention smoking prevalence and selected structural covariates measured in 2013. These covariates were chosen to capture key macro-level determinants of tobacco use in low- and middle-income countries, including socioeconomic status, educational attainment, and access to health services. Specifically, human development index and poverty rate proxy broader socioeconomic conditions; literacy and school enrollment reflect educational attainment and youth exposure to health information; and health facility density captures access to healthcare, including prevention and cessation services.^23^^,^^24^

Matching on these structural characteristics, in addition to baseline smoking prevalence, improves the plausibility of the counterfactual by aligning both the level and underlying determinants of smoking behavior between Bogor City and its synthetic control. This approach is consistent with established guidance, which emphasizes the importance of combining pre-intervention outcomes with theoretically relevant predictors to reduce bias from structural differences that may influence outcome trajectories.^15^

All analyses were conducted using Stata 15 (StataCorp LLC, College Station, TX, USA).

### Ethics statement

The ethical assessment procedure for this research was conducted by the Research and Community Engagement Ethical Committee at the Faculty of Public Health, Universitas Indonesia, and approval was granted with ethical approval number 648/UN2.F10.D11/PPM.00.02/2023.

### Role of the funding source

The funder had no role in study design, data collection and analysis/interpretation, or preparation of the manuscript.

## Results

Table 1 presents the mean values of baseline predictors and outcome variables in 2013 for both the treated unit (Bogor City) and its synthetic control. The treated and synthetic units were closely matched on key pre-intervention characteristics. The human development index was nearly identical (66.7 vs. 66.8), and literacy rates and school completion rates—particularly for primary (97.9% vs. 96.0%) and junior high school (79.1% vs. 70.6%)—showed relatively good balance, although senior high school completion was higher in Bogor (62.1%) than in the synthetic control (46.0%). The poverty rate was slightly lower in Bogor (9.5% vs. 11.7%), while health service availability differed somewhat (health centers: 0.1 vs. 1.4 per 1000 population; hospitals: 0.7 vs. 1.6 per 1000 population). Pre-treatment smoking prevalence in 2013 was nearly identical: ever smoker and current smoker rates among boys were 30.0% and 24.8% in Bogor and in the synthetic control, while among adult men, ever smoking was 88.3% vs. 89.1% and current smoking was 78.6% vs. 75.6%. These results demonstrate strong baseline comparability between Bogor and its synthetic counterpart prior to the intervention.

**Table 1:** Mean values of predictors at the pre-treatment period (2013).

Predictor	Treated	Synthetic
(a) Common predictors (2013)		
Human development index (IPM)	66.7	66.8
Poverty rate (%)	9.5	11.7
Literacy rate (%)	96.8	93.7
Primary school completion (%)	97.9	96.0
Junior high school completion (%)	79.1	70.6
Senior high school completion (%)	62.1	46.0
Health centers (per 1000 pop)	0.1	1.4
Hospitals (per 1000 pop)	0.7	1.6
(b) Outcome variable (2013)		
Ever smoker: Boys (%)	30.0	30.0
Current smoker: Boys (%)	24.8	24.8
Ever smoker: Men (%)	88.3	89.1
Current smoker: Men (%)	78.6	75.6

Note: This table summarizes the balance between treated and synthetic Kota Bogor on all predictors used in the synthetic control models. Common predictors—such as human development index, poverty rate, education levels, and health facility density—were included in all models. Additionally, the 2013 prevalence of each outcome variable (e.g., ever smoker among boys) was included as a predictor in its respective model. All values reflect pre-intervention characteristics.

Table 2 lists the top contributing districts and their weights in the synthetic control models, with the full list provided in Appendices S1A–D. The synthetic controls were constructed from a weighted combination of 240 donor districts, with the top 10 contributors for each outcome listed. Temanggung consistently received the highest weight in three of four outcomes—ever smoker boys (0.584), current smoker boys (0.675), and ever smoker men (0.713)—indicating close alignment with Bogor City's pre-intervention characteristics. For current smoker men, Pagar Alam contributed the largest weight (0.343), followed by Temanggung (0.174) and Bener Meriah (0.093). While top contributors varied slightly across outcomes, the models consistently drew from districts with similar socioeconomic and health system profiles, reflecting robustness in the synthetic control construction.

**Table 2:** Country weights in the synthetic control districts for reproducing ever smokers and current smokers trends in Bogor City, 2013–2023.

Rank	Ever smokers: Boys		Current smokers: Boys		Ever smokers: Men		Current smokers: Men	
	District	Weight	District	Weight	District	Weight	District	Weight
1	Temanggung	0.584	Temanggung	0.675	Temanggung	0.713	Pagar Alam	0.343
2	Magelang	0.007	Pagar Alam	0.005	Pagar Alam	0.004	Temanggung	0.174
3	Malang	0.007	Malang	0.005	Magelang	0.004	Bener Meriah	0.093
4	Tana Toraja	0.006	Bitung	0.005	Bener Meriah	0.003	Ciamis	0.036
5	Pagar Alam	0.005	Tasikmalaya	0.004	Karo	0.003	Bantaeng	0.016
6	Bekasi	0.005	Gunung Mas	0.004	Samosir	0.003	Karo	0.007
7	Tasikmalaya	0.005	Kutai Barat	0.004	Solok	0.003	Dairi	0.002
8	Semarang	0.005	Tomohon	0.004	Lima Puluh Kota	0.003	Aceh Singkil	0.001
9	Pacitan	0.005	Tana Toraja	0.004	Solok	0.003	Aceh Tenggara	0.001
10	Bitung	0.005	Lhokseumawe	0.003	Bogor	0.003	Aceh Tengah	0.001

Note: Kota Bogor's synthetic control was constructed from a weighted average of 240 donor districts. This table presents the top 10 contributors for each outcome variable; the full list of weights is provided in Appendices 1A–D.

**Fig. 1:**
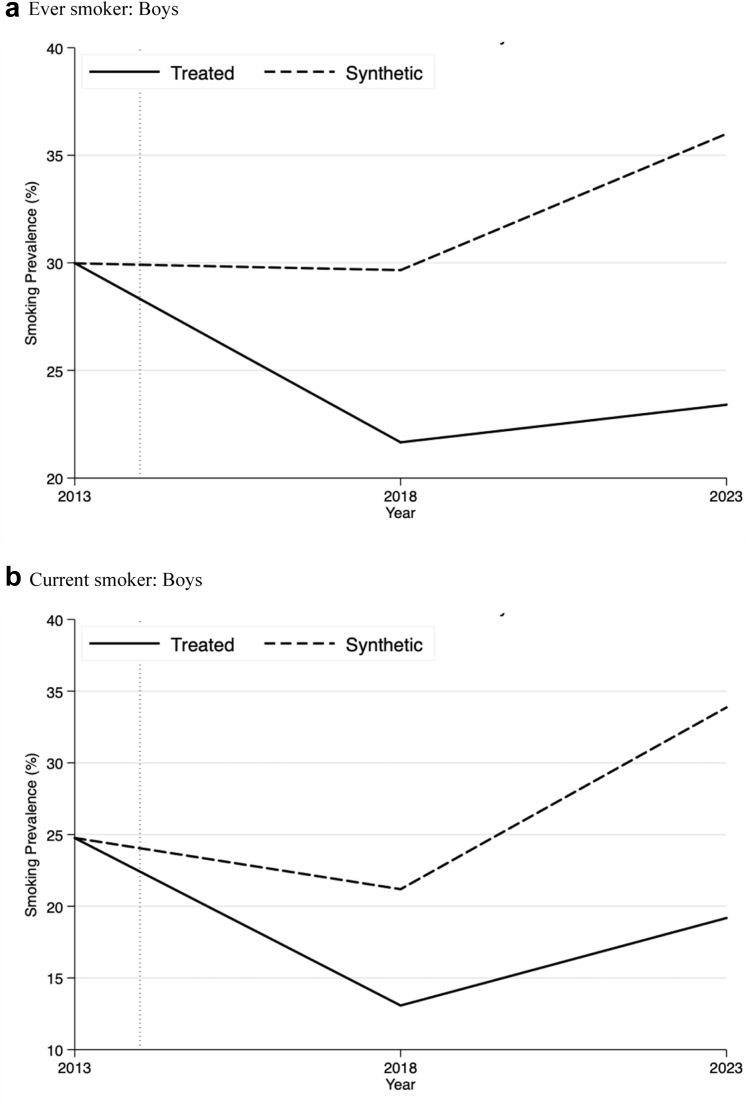
**Trends in smoking prevalence among boys: Bogor City versus its synthetic control (2013–2023).** (a) Ever smoker: Boys. (b) Current smoker: Boys. Note: Each panel compares the observed smoking prevalence in Bogor City (solid line) with its synthetic control (dashed line) constructed from a weighted combination of comparison districts. Panel (a) shows ever smoking prevalence, and panel (b) shows current smoking prevalence. The vertical dotted line marks 2014, the year of implementation of the tobacco advertising, promotion, and sponsorship (TAPS) ban in Bogor City. Smoking prevalence is observed in 2013 (pre-intervention), 2018 (first post-intervention observation), and 2023.

**Fig. 2:**
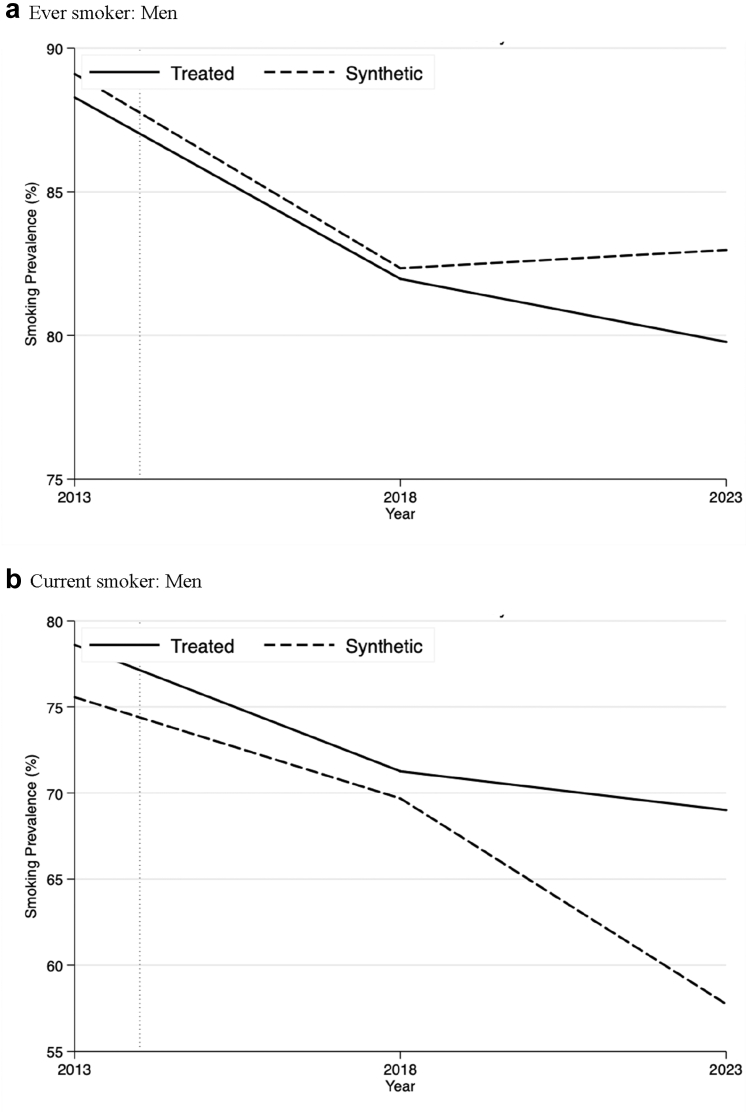
**Trends in smoking prevalence among men: Bogor City versus its synthetic control district (2013 2023).** (a) Ever smoker: Men. (b) Current smoker: Men. Note: Each panel compares the observed smoking prevalence in Bogor City (solid line) with its synthetic control (dashed line) constructed from a weighted combination of comparison districts. Panel (a) shows ever smoking prevalence, and panel (b) shows current smoking prevalence. The vertical dotted line marks 2014, the year of implementation of the tobacco advertising, promotion, and sponsorship (TAPS) ban in Bogor City. Smoking prevalence is observed in 2013 (pre-intervention), 2018 (first post-intervention observation), and 2023.

Figs. 1 and 2, together with Table 3, summarize the estimated effects of Bogor City's comprehensive tobacco control policy from 2013 to 2023. The most pronounced impact was among male adolescents. For ever smokers, prevalence in Bogor declined from 30.0% in 2013 to 23.4% in 2023, while the synthetic control rose to 35.7%, corresponding to a 12.3 percentage point reduction and a 34.5% relative reduction. For current smokers, prevalence fell from 24.8% to 19.2% in Bogor while rising to 33.9% in the synthetic control—corresponding to a 14.7 percentage point reduction and a 43.4% relative reduction. These findings demonstrate strong and sustained policy effects in reducing both smoking initiation and ongoing tobacco use among adolescent boys.

**Table 3:** Actual estimates of treatment effects.

Outcome	Year	Treated (%)	Synthetic (%)	Absolute Diff (pp)	Relative Diff (%)
	[1]	[2]	[3]	[4] = [2]−[3]	[5] = [4]/[3]
Ever smoker: Boys	2013	30.0	30.0	0.0	0.1
	2018	21.7	29.7	−8.0	−27.0
	2023	23.4	35.7	−12.3	−34.5
Current smoker: Boys	2013	24.8	24.8	−0.0	−0.04
	2018	13.1	21.2	−8.1	−38.3
	2023	19.2	33.9	−14.7	−43.4
Ever smoker: Adult Men	2013	88.3	89.1	−0.8	−0.9
	2018	82.0	82.3	−0.4	−0.5
	2023	79.8	83.0	−3.2	−3.9
Current smoker: Adult Men	2013	78.6	75.6	3.1	4.0
	2018	71.3	69.7	1.6	2.3
	2023	69.0	57.7	11.3	19.5

Note: Diff = Difference, Pp = percentage points. Treatment effects in column [4] are calculated by subtracting the synthetic control's smoking prevalence from the observed prevalence in the treated unit (Kota Bogor). Column [5] expresses this difference as a percentage of the synthetic control value. The synthetic control values were derived by applying donor district weights (see Table 2) to the smoking prevalence rates of contributing districts in the donor pool.

Effects among adult men were smaller and mixed. Ever smoking declined modestly in Bogor City from 88.3% to 79.8%, compared with 89.1%–83.0% in the synthetic control, corresponding to a 3.2 percentage point difference (3.9% relative reduction) in 2023. For current smoking, Bogor underperformed its synthetic control: prevalence declined from 78.6% to 69.0% in Bogor versus 57.7% in the synthetic control, leaving prevalence 11.3 percentage points higher in Bogor, corresponding to a 19.5% relative difference in 2023.

The robustness check using all districts as the donor pool supports the main findings (Appendix 2). The magnitude and direction of effects remained consistent with the original synthetic control analysis. Among adolescent boys, Bogor continued to show substantial reductions in both ever and current smoking rates relative to the synthetic control, with relative reductions of 35.1% in 2023 for both outcomes, closely mirroring the original estimates of 34.5% and 43.4%, respectively. Among adult men, effects were again modest, and the gap in current smoking prevalence narrowed slightly (from 11.3 to 5.6 percentage points in 2023). These results confirm that the main conclusions are not sensitive to donor pool selection.

**Fig. 3:**
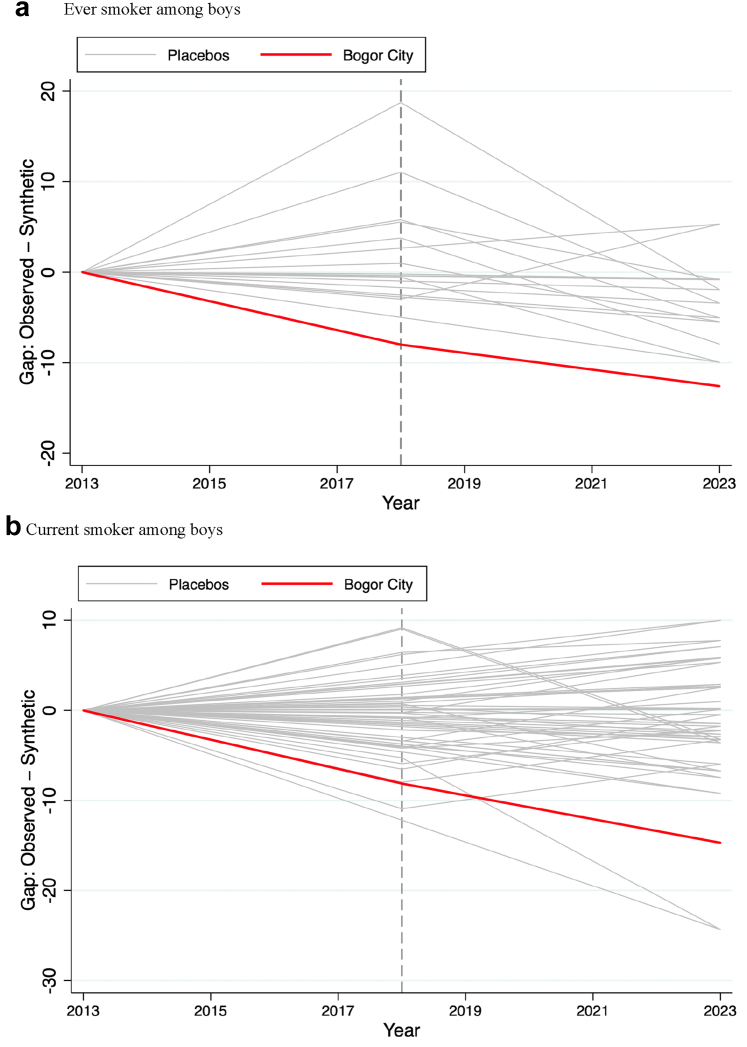
**Placebo tests for adolescent smoking outcomes: (a) ever smoker and (b) current smoker among boys (a) Ever smoker among boys.** (b) Current smoker among boys. Note: Each gray line represents the gap (observed minus synthetic prevalence) for a placebo district, while the red line represents Bogor City. The vertical dashed line corresponds to 2018, the first post-intervention survey wave. The tobacco advertising, promotion, and sponsorship (TAPS) ban was implemented in 2014; with 2013 representing the pre-intervention baseline.

**Fig. 4:**
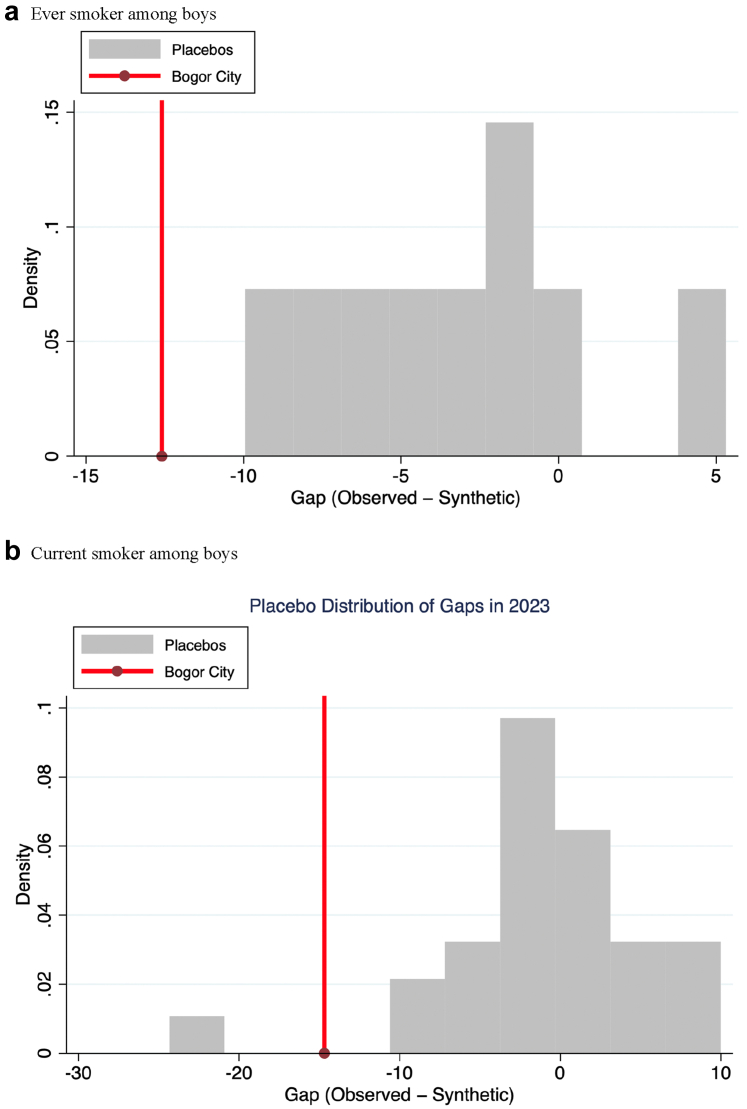
**Placebo distribution of post-intervention gaps in 2023 for ever smoking prevalence among boys.** (a) Ever smoker among boys. (b) Current smoker among boys. Note: This figure shows the distribution of post-intervention gaps (observed minus synthetic prevalence) in 2023 for placebo districts that did not implement comprehensive tobacco control policies (gray bars). The red vertical line marks Bogor City's observed gap. The tobacco advertising, promotion, and sponsorship (TAPS) ban was implemented in 2014, with 2018 representing the first post-intervention observation based on available survey data. Pseudo p-values were calculated as the proportion of placebo districts with absolute gaps greater than or equal to Bogor City's gap, limited to districts with good pre-intervention fit. None of the nine placebo districts had a gap as large as Bogor City for ever smoker boys (pseudo p-value <0.001), and only one did for current smoker boys (pseudo p-value = 0.037), suggesting the observed effects are unlikely to have occurred by chance.

Figs. 3 and 4 present the placebo test results for adolescent smoking outcomes. In the time-series placebo analysis (Fig. 3), Bogor City exhibited a substantially larger post-policy reduction in ever smoking prevalence among boys compared with all placebo districts, and a greater decline in current smoking prevalence than nearly all placebo districts. The placebo gap distributions (Fig. 4) further confirm these findings. For ever smokers, Bogor's 2023 gap (12.6 percentage points lower than the synthetic control) was more extreme than any placebo district (pseudo p-value <0.001), while for current smokers, only one placebo district had an equal or greater gap (pseudo p-value = 0.037). These results indicate that the observed reductions in adolescent smoking prevalence in Bogor are unlikely to have occurred by chance.

## Discussion

This study provides empirical evidence on the effects of district-level comprehensive tobacco control in Indonesia, using Bogor City as a case study. By employing the synthetic control method and leveraging nationally representative survey data across a decade, we found that Bogor's 2014 ban on TAPS—along with associated tobacco control efforts—was associated with substantial reductions in smoking prevalence among male adolescents. These reductions were both statistically and substantively meaningful, with relative declines exceeding 40% for current smoking and 30% for ever smoking by 2023 compared to a synthetic counterfactual. These findings indicate that well-enforced local advertising and promotion restrictions can meaningfully alter youth smoking trajectories even in a permissive national regulatory environment.

Although the 2014 outdoor TAPS ban was used as the intervention point in the synthetic control model, Bogor City's tobacco control efforts were cumulative and sequential, reflecting a series of progressively strengthened policies rather than a single policy change or one-time intervention. The city adopted a comprehensive smoke-free law in 2009, strengthened enforcement through mayoral regulations and mobile court inspections in 2010, and later introduced a point-of-sale display ban in 2017.^12^, ^13^, ^14^ The 2014 regulation represented a significant intensification and expansion of advertising restrictions rather than the onset of tobacco control activity. Importantly, Bogor's policy trajectory was supported by strong political commitment, routine enforcement through inspections, public awareness campaigns, and active resistance to tobacco industry interference, factors that likely reinforced policy effectiveness and compliance.^25^ Consequently, the observed reductions likely reflect the cumulative impact of progressively strengthened local policies embedded within a supportive institutional and political context, rather than the isolated effect of a single regulatory act.

In contrast, the effects among adult men were more modest, particularly for current smoking prevalence, which declined more rapidly in the synthetic control than in Bogor City. This relative pattern highlights the differentiated responsiveness of youth and adult populations to subnational tobacco control interventions and suggests that policies effective for preventing initiation may not generate equivalent effects for cessation among established smokers.

Our findings were robust across alternative specifications and placebo tests. Re-estimating the synthetic control model using the full donor pool produced treatment effects that closely mirrored our main estimates, indicating that results were not sensitive to donor pool selection. Placebo analyses further demonstrated that the observed reductions in ever and current smoking among boys in Bogor City were unlikely to have occurred by chance, with none of the placebo districts showing a gap as large as Bogor City for ever smokers and only one showing a similar gap for current smokers. These robustness checks strengthen confidence in our conclusion that Bogor City's tobacco control policies were associated with meaningful reductions in adolescent smoking prevalence.

The widening divergence between Bogor City and its synthetic control after 2018 was partly driven by an upward trend in smoking prevalence among donor districts between 2018 and 2023. Several contextual factors may explain this pattern. First, tobacco control enforcement outside pioneering districts such as Bogor may have remained limited, reflecting Indonesia's decentralized governance structure and documented heterogeneity in local policy implementation and compliance,^10^^,^^26^, ^27^, ^28^ as well as substantial regional variation in smoking prevalence across districts.^29^^,^^30^ Second, broader structural changes—including economic shifts and evolving tobacco marketing and regulatory dynamics in Indonesia^31^^,^^32^—alongside disruptions related to the COVID-19 pandemic, may have differentially influenced youth smoking behaviors across districts. Although Riskesdas surveys use consistent national methodology, minor differences in implementation or sampling across waves could have influenced observed trends. Sensitivity analyses excluding influential donor districts yielded similar estimates, indicating that the divergence reflected broader comparison-pool dynamics rather than a single outlier. Nevertheless, the limited number of survey waves warrants cautious interpretation.

The decline observed in the synthetic control for adult men between 2013 and 2023 suggests that smoking prevalence declined across many comparison districts during this period, likely reflecting broader secular trends rather than the absence of intervention. National-level developments—including gradual tobacco excise tax increases, expansion of pictorial health warnings, and shifting social norms—may have contributed to reductions in adult smoking across Indonesia.^6^^,^^7^^,^^33^ Economic transitions and increasing health awareness may also have supported cessation behaviors in districts beyond Bogor, while diffusion effects from smoke-free policies adopted in other jurisdictions may have generated spillover reductions independent of Bogor's 2014 TAPS ban.^20^^,^^21^

These broader trends are important for interpreting Bogor City's more modest relative performance in current smoking among adult men, as they suggest that declines in the synthetic control reflect wider contextual changes rather than the absence of policy impact in Bogor itself. Against this backdrop, Bogor's more modest reduction in current smoking likely reflects the limited direct impact of advertising restrictions on established adult smokers, for whom cessation typically requires price measures, sustained enforcement, and structured cessation support.^15^, ^16^, ^17^ Importantly, this finding reflects a relative comparison with the synthetic control. The divergence therefore appears driven by secular declines in adult smoking across comparison districts, combined with the limited capacity of advertising restrictions alone to generate substantial cessation among established smokers.

Taken together, these findings suggest that district-level advertising and promotion restrictions may be particularly effective in preventing youth initiation but insufficient, on their own, to substantially reduce smoking among established adult users. This distinction is consistent with tobacco control theory, which emphasizes that different components of the MPOWER framework operate through distinct behavioral pathways: restrictions on advertising and promotion primarily limit exposure and initiation among adolescents,^9^^,^^34^^,^^35^ whereas price measures, cessation services, and sustained enforcement are more central to adult quitting.^6^^,^^7^^,^^15^ In this sense, Bogor City's experience illustrates both the strengths and limits of subnational policy action when implemented in the absence of comprehensive national measures such as substantial excise taxation and integrated cessation support.^6^^,^^7^ The findings therefore reinforce the importance of layered tobacco control strategies rather than reliance on a single policy instrument.^5^^,^^17^

Within the broader MPOWER framework, these results underscore the importance of integrating demand-reduction policies with preventive and educational interventions. Advertising and promotion bans may reduce exposure and initiation risk, but sustained reductions in youth smoking likely require complementary measures such as school-based tobacco prevention programs, mass media campaigns, and consistent enforcement of smoke-free environments.^36^ Recent analysis of Global Youth Tobacco Survey data across 38 LMICs found that only 59% of adolescents reported exposure to school-based tobacco prevention education, highlighting substantial gaps in preventive coverage.^37^ Strengthening school-based interventions alongside policy measures could amplify and sustain the gains observed in Bogor City, particularly in decentralized systems where local governments can shape both regulatory and educational environments.

Our findings align with international evidence on the effectiveness of local tobacco control efforts in contexts where national-level enforcement is weak. For instance, a study from Thailand found substantial reductions in youth smoking following subnational tobacco-free school zone policies,^16^ while South Africa's national tax-led tobacco control strategy yielded a 36% reduction in per capita consumption over a decade.^15^ However, Indonesia presents a distinct policy context: it is among a handful of countries that has not ratified the FCTC, and national implementation of MPOWER measures remains limited.^5^^,^^7^^,^^38^ As such, district-level initiatives like Bogor City's TAPS ban represent an important yet underutilized avenue for tobacco control in Indonesia's decentralized governance system.^26^, ^27^, ^28^ Our findings provide the first quantitative evidence supporting their effectiveness in this setting.

The observed adolescent smoking reductions may reflect the heightened visibility and enforceability of TAPS restrictions in environments where youth are exposed to pervasive tobacco advertising and branded promotions.^9^^,^^31^^,^^34^^,^^35^^,^^39^ In contrast, the lack of observed benefits—and potential adverse trend—for current smoking among adult men may reflect limited cessation support, continued exposure to tobacco sales and availability, or policy spillovers from neighboring areas.^11^^,^^19^^,^^33^ Prior studies have highlighted the challenges of adult smoking reduction in environments where cigarette affordability remains high and policy enforcement uneven.^6^^,^^7^^,^^40^^,^^41^

This study offers timely and actionable insights for policymakers in Indonesia and other LMICs with similar institutional and tobacco industry challenges. The significant reductions in adolescent smoking observed in Bogor City—achieved through locally enacted comprehensive tobacco control measures—underscore the potential of subnational leadership in advancing public health.^6^^,^^10^^,^^14^ These findings suggest that subnational initiatives can generate measurable public health gains even in weak national environments, but their long-term impact may be constrained without complementary national-level measures such as meaningful excise tax increases, comprehensive marketing restrictions, and accessible cessation support.^6^^,^^7^^,^^38^ To put this into perspective, neighboring countries such as Thailand and the Philippines, with comparable development contexts, ratified the FCTC two decades ago. Indonesia remains one of the few countries yet to do so, despite facing a growing burden of tobacco-related health and social consequences.^1^^,^^2^^,^^38^ Ratification would not only help reduce smoking-related health burdens but also generate broader societal benefits, including improved educational attainment, household financial stability, and workforce productivity.

This study highlights that even amid weak national tobacco control enforcement, local governments can play a critical leadership role.^32^ Bogor City's success demonstrates that, despite limited national commitment, strong and well-enforced local policies can deliver substantial public health benefits.^13^^,^^26^^,^^28^ This should serve as a wake-up call for the national government to intensify its efforts and establish a more robust and supportive policy environment. For districts committed to improving the health, educational attainment, and economic potential of their youth, Bogor offers a replicable model. Empowering more local jurisdictions to implement comprehensive tobacco control can help bridge national policy gaps and accelerate progress toward equitable and sustainable development.

Interpretation of these findings should be considered in light of several limitations. First, smoking prevalence was observed at only one pre-intervention time point (2013), which precluded formal modeling of pre-policy trends. Consequently, we cannot exclude the possibility that some of the post-2014 reductions reflect continuation of underlying secular trends rather than solely the effect of Bogor City's tobacco control measures. Although the synthetic control method improves comparability by matching on baseline prevalence and structural covariates, its ability to replicate pre-intervention trajectories was necessarily constrained by the available data.

Second, smoking among women and girls was not examined due to low prevalence and resulting statistical instability at the district level. While this analytic decision was necessary for robust subnational modeling, it limits the generalizability of findings to girls and women. Although female smoking prevalence in Indonesia remains substantially lower than among males, gender norms, targeted marketing strategies, and evolving social patterns may influence tobacco uptake differently across sexes. Future research should examine whether subnational tobacco control policies have differential effects by gender, particularly as tobacco industry marketing increasingly targets young women in emerging markets.

Third, our analysis relies on aggregate ecological data, which may mask individual-level heterogeneity in smoking behaviors. Fourth, while we controlled for key socioeconomic predictors, unobserved time-varying confounders may still bias estimates. Finally, we were unable to directly evaluate enforcement quality, concurrent policies, or tobacco industry interference at the local level, all of which could modulate policy effectiveness.^6^^,^^10^^,^^27^ Future research integrating qualitative and process evaluation approaches could provide deeper insight into how political leadership, enforcement practices, institutional capacity, and resistance to tobacco industry interference shaped policy implementation and effectiveness in Bogor and other districts.

Despite these limitations, the synthetic control framework enables a structured and transparent counterfactual comparison, and the findings contribute meaningful evidence on the potential role of subnational tobacco control in a decentralized policy environment.

In conclusion. This study demonstrates that strong local tobacco control policies can achieve reductions in adolescent smoking, even in settings with weak national enforcement. Using a synthetic control approach, we found that Bogor City's progressively strengthened tobacco control measures, intensified from 2014 onward, were associated with declines in both ever and current smoking among male adolescents—by up to 43% relative to the synthetic control—while impacts among adult men were modest or mixed. These findings underscore the effectiveness of preventive strategies targeting youth and highlight the potential of local leadership in advancing tobacco control. As Indonesia remains one of the few countries yet to ratify the FCTC, national policymakers should consider strengthening their commitment to protecting future generations. In parallel, local governments across Indonesia can draw on Bogor City's experience to accelerate public health gains, reduce long-term economic burdens, and safeguard population health and development.

## Contributors

DK conceived the study. HM led data collection and cleaning. DK conducted the analyses. DK drafted the manuscript, and HM contributed to revisions. All authors read and approved the final manuscript.

## Data sharing statement

Available from the authors upon reasonable request.

## Declaration of interests

The authors declare no competing interests.
