# Supplementary material for: Impact of district-level comprehensive tobacco control on smoking among adolescent boys in Indonesia: a synthetic control study (2013–2023)
Source: Lancet Reg Health Southeast Asia. 2026 Jun 11;50:100798. doi: 10.1016/j.lansea.2026.100798 (PMC13276449; doi:10.1016/j.lansea.2026.100798)
Supplement: Supplementary Material [file mmc1.docx]

**Appendix 1A. Weights for ever smokers among boys**

| No | District | Weight | No | District | Weight | No | District | Weight | No | District | Weight |
| --- | --- | --- | --- | --- | --- | --- | --- | --- | --- | --- | --- |
| 1 | TEMANGGUNG | 0.584 | 61 | LUBUKLINGGAU | 0.002 | 121 | SOLOK SELATAN | 0.001 | 181 | TUBAN | 0.001 |
| 2 | MAGELANG | 0.007 | 62 | REJANG LEBONG | 0.002 | 122 | DHARMASRAYA | 0.001 | 182 | LAMONGAN | 0.001 |
| 3 | MALANG | 0.007 | 63 | KARIMUN | 0.002 | 123 | PASAMAN BARAT | 0.001 | 183 | GRESIK | 0.001 |
| 4 | TANA TORAJA | 0.006 | 64 | KEPULAUAN ANAMBAS | 0.002 | 124 | PADANG | 0.001 | 184 | BLITAR | 0.001 |
| 5 | PAGAR ALAM | 0.005 | 65 | BATAM | 0.002 | 125 | BUKITTINGGI | 0.001 | 185 | MALANG | 0.001 |
| 6 | BEKASI | 0.005 | 66 | CIAMIS | 0.002 | 126 | PAYAKUMBUH | 0.001 | 186 | PASURUAN | 0.001 |
| 7 | TASIKMALAYA | 0.005 | 67 | KUNINGAN | 0.002 | 127 | PARIAMAN | 0.001 | 187 | SURABAYA | 0.001 |
| 8 | SEMARANG | 0.005 | 68 | SUBANG | 0.002 | 128 | KUANTAN SINGINGI | 0.001 | 188 | TANGERANG | 0.001 |
| 9 | PACITAN | 0.005 | 69 | BANYUMAS | 0.002 | 129 | INDRAGIRI HULU | 0.001 | 189 | MATARAM | 0.001 |
| 10 | BITUNG | 0.005 | 70 | SURAKARTA | 0.002 | 130 | SIAK | 0.001 | 190 | BIMA | 0.001 |
| 11 | SAWAH LUNTO | 0.004 | 71 | SALATIGA | 0.002 | 131 | KAMPAR | 0.001 | 191 | SINGKAWANG | 0.001 |
| 12 | BOGOR | 0.004 | 72 | TRENGGALEK | 0.002 | 132 | ROKAN HULU | 0.001 | 192 | KOTAWARINGIN BARAT | 0.001 |
| 13 | PURBALINGGA | 0.004 | 73 | BLITAR | 0.002 | 133 | BENGKALIS | 0.001 | 193 | BARITO SELATAN | 0.001 |
| 14 | SLEMAN | 0.004 | 74 | KEDIRI | 0.002 | 134 | ROKAN HILIR | 0.001 | 194 | LAMANDAU | 0.001 |
| 15 | GUNUNG MAS | 0.004 | 75 | BANYUWANGI | 0.002 | 135 | DUMAI | 0.001 | 195 | BARITO TIMUR | 0.001 |
| 16 | BALANGAN | 0.004 | 76 | SIDOARJO | 0.002 | 136 | KERINCI | 0.001 | 196 | TANAH LAUT | 0.001 |
| 17 | KUTAI BARAT | 0.004 | 77 | MOJOKERTO | 0.002 | 137 | MERANGIN | 0.001 | 197 | KATINGAN | 0.001 |
| 18 | TOMOHON | 0.004 | 78 | MOJOKERTO | 0.002 | 138 | BATANG HARI | 0.001 | 198 | KOTABARU | 0.001 |
| 19 | PINRANG | 0.004 | 79 | BATU | 0.002 | 139 | MUARO JAMBI | 0.001 | 199 | BANJAR | 0.001 |
| 20 | LHOKSEUMAWE | 0.003 | 80 | TANGERANG | 0.002 | 140 | TEBO | 0.001 | 200 | TAPIN | 0.001 |
| 21 | PADANG LAWAS | 0.003 | 81 | CILEGON | 0.002 | 141 | BUNGO | 0.001 | 201 | HULU SUNGAI SELATAN | 0.001 |
| 22 | LIMA PULUH KOTA | 0.003 | 82 | JEMBRANA | 0.002 | 142 | JAMBI | 0.001 | 202 | HULU SUNGAI TENGAH | 0.001 |
| 23 | PRABUMULIH | 0.003 | 83 | BANGLI | 0.002 | 143 | OGAN KOMERING ULU | 0.001 | 203 | TABALONG | 0.001 |
| 24 | BENGKULU SELATAN | 0.003 | 84 | LOMBOK BARAT | 0.002 | 144 | OGAN KOMERING ULU TIMUR | 0.001 | 204 | TANAH BUMBU | 0.001 |
| 25 | PANGKALPINANG | 0.003 | 85 | MINAHASA | 0.002 | 145 | PALEMBANG | 0.001 | 205 | BANJARMASIN | 0.001 |
| 26 | NATUNA | 0.003 | 86 | MINAHASA TENGGARA | 0.002 | 146 | BENGKULU UTARA | 0.001 | 206 | BANJAR BARU | 0.001 |
| 27 | BANDUNG | 0.003 | 87 | KOTAMOBAGU | 0.002 | 147 | BENGKULU | 0.001 | 207 | KUTAI TIMUR | 0.001 |
| 28 | PURWAKARTA | 0.003 | 88 | BANGGAI | 0.002 | 148 | LAMPUNG TIMUR | 0.001 | 208 | BERAU | 0.001 |
| 29 | SUKABUMI | 0.003 | 89 | POSO | 0.002 | 149 | PRINGSEWU | 0.001 | 209 | BALIKPAPAN | 0.001 |
| 30 | CIMAHI | 0.003 | 90 | WAJO | 0.002 | 150 | BELITUNG | 0.001 | 210 | BONTANG | 0.001 |
| 31 | BANJAR | 0.003 | 91 | SIDENRENG RAPPANG | 0.002 | 151 | BANGKA TENGAH | 0.001 | 211 | MALINAU | 0.001 |
| 32 | KEBUMEN | 0.003 | 92 | WAKATOBI | 0.002 | 152 | BINTAN | 0.001 | 212 | BULUNGAN | 0.001 |
| 33 | BOYOLALI | 0.003 | 93 | GORONTALO | 0.002 | 153 | TANJUNG PINANG | 0.001 | 213 | TARAKAN | 0.001 |
| 34 | MAGELANG | 0.003 | 94 | AMBON | 0.002 | 154 | CIREBON | 0.001 | 214 | KEPULAUAN SANGIHE | 0.001 |
| 35 | BANTUL | 0.003 | 95 | ACEH SINGKIL | 0.001 | 155 | SUMEDANG | 0.001 | 215 | MANADO | 0.001 |
| 36 | GUNUNG KIDUL | 0.003 | 96 | ACEH TENGGARA | 0.001 | 156 | KARAWANG | 0.001 | 216 | PALU | 0.001 |
| 37 | YOGYAKARTA | 0.003 | 97 | ACEH TENGAH | 0.001 | 157 | BEKASI | 0.001 | 217 | GOWA | 0.001 |
| 38 | TULUNGAGUNG | 0.003 | 98 | ACEH BESAR | 0.001 | 158 | BANDUNG | 0.001 | 218 | MAROS | 0.001 |
| 39 | MAGETAN | 0.003 | 99 | PIDIE | 0.001 | 159 | CIREBON | 0.001 | 219 | PANGKAJENE DAN KEPULAUAN | 0.001 |
| 40 | NGAWI | 0.003 | 100 | ACEH UTARA | 0.001 | 160 | CILACAP | 0.001 | 220 | BARRU | 0.001 |
| 41 | PROBOLINGGO | 0.003 | 101 | ACEH TAMIANG | 0.001 | 161 | PURWOREJO | 0.001 | 221 | SOPPENG | 0.001 |
| 42 | SERANG | 0.003 | 102 | NAGAN RAYA | 0.001 | 162 | KLATEN | 0.001 | 222 | ENREKANG | 0.001 |
| 43 | MOROWALI | 0.003 | 103 | ACEH JAYA | 0.001 | 163 | WONOGIRI | 0.001 | 223 | LUWU UTARA | 0.001 |
| 44 | BULUKUMBA | 0.003 | 104 | BANDA ACEH | 0.001 | 164 | KARANGANYAR | 0.001 | 224 | TORAJA UTARA | 0.001 |
| 45 | BANTAENG | 0.003 | 105 | LANGSA | 0.001 | 165 | GROBOGAN | 0.001 | 225 | PAREPARE | 0.001 |
| 46 | LUWU | 0.003 | 106 | TAPANULI SELATAN | 0.001 | 166 | BLORA | 0.001 | 226 | MUNA | 0.001 |
| 47 | MAKASSAR | 0.003 | 107 | LABUHAN BATU | 0.001 | 167 | JEPARA | 0.001 | 227 | KOLAKA | 0.001 |
| 48 | PALOPO | 0.003 | 108 | SIMALUNGUN | 0.001 | 168 | DEMAK | 0.001 | 228 | KONAWE SELATAN | 0.001 |
| 49 | MIMIKA | 0.003 | 109 | DAIRI | 0.001 | 169 | BATANG | 0.001 | 229 | KOLAKA UTARA | 0.001 |
| 50 | BIREUEN | 0.002 | 110 | KARO | 0.001 | 170 | PEKALONGAN | 0.001 | 230 | BAUBAU | 0.001 |
| 51 | BENER MERIAH | 0.002 | 111 | LANGKAT | 0.001 | 171 | TEGAL | 0.001 | 231 | BONE BOLANGO | 0.001 |
| 52 | PIDIE JAYA | 0.002 | 112 | SAMOSIR | 0.001 | 172 | SEMARANG | 0.001 | 232 | MALUKU TENGAH | 0.001 |
| 53 | DELI SERDANG | 0.002 | 113 | SERDANG BEDAGAI | 0.001 | 173 | PEKALONGAN | 0.001 | 233 | BURU | 0.001 |
| 54 | TEBING TINGGI | 0.002 | 114 | BATU BARA | 0.001 | 174 | TEGAL | 0.001 | 234 | TERNATE | 0.001 |
| 55 | SOLOK | 0.002 | 115 | PADANG LAWAS UTARA | 0.001 | 175 | PONOROGO | 0.001 | 235 | TIDORE KEPULAUAN | 0.001 |
| 56 | TANAH DATAR | 0.002 | 116 | TANJUNG BALAI | 0.001 | 176 | PASURUAN | 0.001 | 236 | MANOKWARI | 0.001 |
| 57 | PADANG PARIAMAN | 0.002 | 117 | PEMATANG SIANTAR | 0.001 | 177 | JOMBANG | 0.001 | 237 | SORONG | 0.001 |
| 58 | AGAM | 0.002 | 118 | MEDAN | 0.001 | 178 | NGANJUK | 0.001 | 238 | MERAUKE | 0.001 |
| 59 | SOLOK | 0.002 | 119 | BINJAI | 0.001 | 179 | MADIUN | 0.001 | 239 | NABIRE | 0.001 |
| 60 | MUARA ENIM | 0.002 | 120 | PESISIR SELATAN | 0.001 | 180 | BOJONEGORO | 0.001 | 240 | JAYAPURA | 0.001 |

**Appendix 1B: Weights for current smokers among boys**

| No | District | Weight | No | District | Weight | No | District | Weight | No | District | Weight |
| --- | --- | --- | --- | --- | --- | --- | --- | --- | --- | --- | --- |
| 1 | TEMANGGUNG | 0.675 | 61 | BANGLI | 0.002 | 121 | PALEMBANG | 0.001 | 181 | BANJAR | 0.001 |
| 2 | PAGAR ALAM | 0.005 | 62 | BALANGAN | 0.002 | 122 | BENGKULU UTARA | 0.001 | 182 | TAPIN | 0.001 |
| 3 | MALANG | 0.005 | 63 | MINAHASA | 0.002 | 123 | BENGKULU | 0.001 | 183 | HULU SUNGAI SELATAN | 0.001 |
| 4 | BITUNG | 0.005 | 64 | MINAHASA TENGGARA | 0.002 | 124 | LAMPUNG TIMUR | 0.001 | 184 | TABALONG | 0.001 |
| 5 | TASIKMALAYA | 0.004 | 65 | KOTAMOBAGU | 0.002 | 125 | PRINGSEWU | 0.001 | 185 | TANAH BUMBU | 0.001 |
| 6 | GUNUNG MAS | 0.004 | 66 | BANGGAI | 0.002 | 126 | BELITUNG | 0.001 | 186 | BANJAR BARU | 0.001 |
| 7 | KUTAI BARAT | 0.004 | 67 | MOROWALI | 0.002 | 127 | BANGKA TENGAH | 0.001 | 187 | KUTAI TIMUR | 0.001 |
| 8 | TOMOHON | 0.004 | 68 | BULUKUMBA | 0.002 | 128 | KARIMUN | 0.001 | 188 | POSO | 0.001 |
| 9 | TANA TORAJA | 0.004 | 69 | BANTAENG | 0.002 | 129 | BINTAN | 0.001 | 189 | GOWA | 0.001 |
| 10 | LHOKSEUMAWE | 0.003 | 70 | PINRANG | 0.002 | 130 | TANJUNG PINANG | 0.001 | 190 | MAROS | 0.001 |
| 11 | TEBING TINGGI | 0.003 | 71 | LUWU | 0.002 | 131 | CIREBON | 0.001 | 191 | BARRU | 0.001 |
| 12 | PRABUMULIH | 0.003 | 72 | PAREPARE | 0.002 | 132 | SUMEDANG | 0.001 | 192 | SOPPENG | 0.001 |
| 13 | BENGKULU SELATAN | 0.003 | 73 | PALOPO | 0.002 | 133 | KARAWANG | 0.001 | 193 | WAJO | 0.001 |
| 14 | PANGKALPINANG | 0.003 | 74 | GORONTALO | 0.002 | 134 | BEKASI | 0.001 | 194 | SIDENRENG RAPPANG | 0.001 |
| 15 | SUBANG | 0.003 | 75 | AMBON | 0.002 | 135 | BANDUNG | 0.001 | 195 | ENREKANG | 0.001 |
| 16 | PURWAKARTA | 0.003 | 76 | MIMIKA | 0.002 | 136 | CIREBON | 0.001 | 196 | LUWU UTARA | 0.001 |
| 17 | SUKABUMI | 0.003 | 77 | ACEH TENGGARA | 0.001 | 137 | CILACAP | 0.001 | 197 | TORAJA UTARA | 0.001 |
| 18 | BEKASI | 0.003 | 78 | ACEH TENGAH | 0.001 | 138 | PURWOREJO | 0.001 | 198 | MUNA | 0.001 |
| 19 | CIMAHI | 0.003 | 79 | ACEH BESAR | 0.001 | 139 | BOYOLALI | 0.001 | 199 | KOLAKA | 0.001 |
| 20 | PURBALINGGA | 0.003 | 80 | PIDIE | 0.001 | 140 | WONOGIRI | 0.001 | 200 | KONAWE SELATAN | 0.001 |
| 21 | PACITAN | 0.003 | 81 | ACEH UTARA | 0.001 | 141 | KARANGANYAR | 0.001 | 201 | WAKATOBI | 0.001 |
| 22 | TULUNGAGUNG | 0.003 | 82 | ACEH TAMIANG | 0.001 | 142 | GROBOGAN | 0.001 | 202 | KOLAKA UTARA | 0.001 |
| 23 | NGAWI | 0.003 | 83 | NAGAN RAYA | 0.001 | 143 | BLORA | 0.001 | 203 | BAUBAU | 0.001 |
| 24 | SERANG | 0.003 | 84 | BENER MERIAH | 0.001 | 144 | JEPARA | 0.001 | 204 | BONE BOLANGO | 0.001 |
| 25 | LOMBOK BARAT | 0.003 | 85 | LANGSA | 0.001 | 145 | DEMAK | 0.001 | 205 | MALUKU TENGAH | 0.001 |
| 26 | MAKASSAR | 0.003 | 86 | TAPANULI SELATAN | 0.001 | 146 | BATANG | 0.001 | 206 | BURU | 0.001 |
| 27 | BIREUEN | 0.002 | 87 | LABUHAN BATU | 0.001 | 147 | PEKALONGAN | 0.001 | 207 | MANOKWARI | 0.001 |
| 28 | ACEH JAYA | 0.002 | 88 | KARO | 0.001 | 148 | TEGAL | 0.001 | 208 | MERAUKE | 0.001 |
| 29 | PIDIE JAYA | 0.002 | 89 | LANGKAT | 0.001 | 149 | SURAKARTA | 0.001 | 209 | NABIRE | 0.001 |
| 30 | DELI SERDANG | 0.002 | 90 | SAMOSIR | 0.001 | 150 | SALATIGA | 0.001 | 210 | JAYAPURA | 0.001 |
| 31 | PADANG LAWAS | 0.002 | 91 | BATU BARA | 0.001 | 151 | SEMARANG | 0.001 |  |  |  |
| 32 | SOLOK | 0.002 | 92 | TANJUNG BALAI | 0.001 | 152 | TEGAL | 0.001 |  |  |  |
| 33 | LIMA PULUH KOTA | 0.002 | 93 | PEMATANG SIANTAR | 0.001 | 153 | BANTUL | 0.001 |  |  |  |
| 34 | PASAMAN BARAT | 0.002 | 94 | MEDAN | 0.001 | 154 | KEDIRI | 0.001 |  |  |  |
| 35 | SOLOK | 0.002 | 95 | BINJAI | 0.001 | 155 | BANYUWANGI | 0.001 |  |  |  |
| 36 | PARIAMAN | 0.002 | 96 | PESISIR SELATAN | 0.001 | 156 | PASURUAN | 0.001 |  |  |  |
| 37 | MUARA ENIM | 0.002 | 97 | TANAH DATAR | 0.001 | 157 | JOMBANG | 0.001 |  |  |  |
| 38 | LUBUKLINGGAU | 0.002 | 98 | PADANG PARIAMAN | 0.001 | 158 | NGANJUK | 0.001 |  |  |  |
| 39 | REJANG LEBONG | 0.002 | 99 | AGAM | 0.001 | 159 | MADIUN | 0.001 |  |  |  |
| 40 | NATUNA | 0.002 | 100 | SOLOK SELATAN | 0.001 | 160 | MAGETAN | 0.001 |  |  |  |
| 41 | BATAM | 0.002 | 101 | DHARMASRAYA | 0.001 | 161 | BOJONEGORO | 0.001 |  |  |  |
| 42 | BOGOR | 0.002 | 102 | PADANG | 0.001 | 162 | TUBAN | 0.001 |  |  |  |
| 43 | BANDUNG | 0.002 | 103 | SAWAH LUNTO | 0.001 | 163 | LAMONGAN | 0.001 |  |  |  |
| 44 | CIAMIS | 0.002 | 104 | BUKITTINGGI | 0.001 | 164 | GRESIK | 0.001 |  |  |  |
| 45 | KUNINGAN | 0.002 | 105 | KUANTAN SINGINGI | 0.001 | 165 | BLITAR | 0.001 |  |  |  |
| 46 | BANJAR | 0.002 | 106 | INDRAGIRI HULU | 0.001 | 166 | MALANG | 0.001 |  |  |  |
| 47 | BANYUMAS | 0.002 | 107 | SIAK | 0.001 | 167 | PASURUAN | 0.001 |  |  |  |
| 48 | KEBUMEN | 0.002 | 108 | KAMPAR | 0.001 | 168 | MOJOKERTO | 0.001 |  |  |  |
| 49 | MAGELANG | 0.002 | 109 | ROKAN HULU | 0.001 | 169 | SURABAYA | 0.001 |  |  |  |
| 50 | SEMARANG | 0.002 | 110 | BENGKALIS | 0.001 | 170 | BATU | 0.001 |  |  |  |
| 51 | MAGELANG | 0.002 | 111 | ROKAN HILIR | 0.001 | 171 | TANGERANG | 0.001 |  |  |  |
| 52 | GUNUNG KIDUL | 0.002 | 112 | DUMAI | 0.001 | 172 | CILEGON | 0.001 |  |  |  |
| 53 | SLEMAN | 0.002 | 113 | KERINCI | 0.001 | 173 | JEMBRANA | 0.001 |  |  |  |
| 54 | YOGYAKARTA | 0.002 | 114 | MERANGIN | 0.001 | 174 | MATARAM | 0.001 |  |  |  |
| 55 | PONOROGO | 0.002 | 115 | BATANG HARI | 0.001 | 175 | BIMA | 0.001 |  |  |  |
| 56 | TRENGGALEK | 0.002 | 116 | TEBO | 0.001 | 176 | LAMANDAU | 0.001 |  |  |  |
| 57 | SIDOARJO | 0.002 | 117 | BUNGO | 0.001 | 177 | BARITO TIMUR | 0.001 |  |  |  |
| 58 | MOJOKERTO | 0.002 | 118 | JAMBI | 0.001 | 178 | TANAH LAUT | 0.001 |  |  |  |
| 59 | PROBOLINGGO | 0.002 | 119 | OGAN KOMERING ULU | 0.001 | 179 | KATINGAN | 0.001 |  |  |  |
| 60 | TANGERANG | 0.002 | 120 | OGAN KOMERING ULU TIMUR | 0.001 | 180 | KOTABARU | 0.001 |  |  |  |

**Appendix 1C: Weights for ever smokers among men**

| No | District | Weight | No | District | Weight | No | District | Weight | No | District | Weight |
| --- | --- | --- | --- | --- | --- | --- | --- | --- | --- | --- | --- |
| 1 | TEMANGGUNG | 0.713 | 61 | PIDIE JAYA | 0.001 | 121 | TANJUNG PINANG | 0.001 | 181 | BARITO TIMUR | 0.001 |
| 2 | PAGAR ALAM | 0.004 | 62 | BANDA ACEH | 0.001 | 122 | KUNINGAN | 0.001 | 182 | KOTABARU | 0.001 |
| 3 | MAGELANG | 0.004 | 63 | SABANG | 0.001 | 123 | KARAWANG | 0.001 | 183 | BANJAR | 0.001 |
| 4 | BENER MERIAH | 0.003 | 64 | LANGSA | 0.001 | 124 | BEKASI | 0.001 | 184 | HULU SUNGAI TENGAH | 0.001 |
| 5 | KARO | 0.003 | 65 | LHOKSEUMAWE | 0.001 | 125 | BEKASI | 0.001 | 185 | TANAH BUMBU | 0.001 |
| 6 | SAMOSIR | 0.003 | 66 | TAPANULI UTARA | 0.001 | 126 | CIMAHI | 0.001 | 186 | BALANGAN | 0.001 |
| 7 | SOLOK | 0.003 | 67 | TOBA | 0.001 | 127 | BANJAR | 0.001 | 187 | PASER | 0.001 |
| 8 | LIMA PULUH KOTA | 0.003 | 68 | LABUHAN BATU | 0.001 | 128 | CILACAP | 0.001 | 188 | KUTAI BARAT | 0.001 |
| 9 | SOLOK | 0.003 | 69 | ASAHAN | 0.001 | 129 | PURWOREJO | 0.001 | 189 | KUTAI KARTANEGARA | 0.001 |
| 10 | BOGOR | 0.003 | 70 | SIMALUNGUN | 0.001 | 130 | BOYOLALI | 0.001 | 190 | BERAU | 0.001 |
| 11 | BANDUNG | 0.003 | 71 | DELI SERDANG | 0.001 | 131 | KLATEN | 0.001 | 191 | SAMARINDA | 0.001 |
| 12 | PURBALINGGA | 0.003 | 72 | LANGKAT | 0.001 | 132 | WONOGIRI | 0.001 | 192 | BONTANG | 0.001 |
| 13 | KEBUMEN | 0.003 | 73 | HUMBANG HASUNDUTAN | 0.001 | 133 | GROBOGAN | 0.001 | 193 | BULUNGAN | 0.001 |
| 14 | BULUKUMBA | 0.003 | 74 | BATU BARA | 0.001 | 134 | BLORA | 0.001 | 194 | TARAKAN | 0.001 |
| 15 | LUWU | 0.003 | 75 | LABUHAN BATU SELATAN | 0.001 | 135 | JEPARA | 0.001 | 195 | MINAHASA | 0.001 |
| 16 | ACEH TENGAH | 0.002 | 76 | SIBOLGA | 0.001 | 136 | BATANG | 0.001 | 196 | KEPULAUAN SANGIHE | 0.001 |
| 17 | BIREUEN | 0.002 | 77 | TANJUNG BALAI | 0.001 | 137 | PEKALONGAN | 0.001 | 197 | KEPULAUAN TALAUD | 0.001 |
| 18 | ACEH JAYA | 0.002 | 78 | PEMATANG SIANTAR | 0.001 | 138 | TEGAL | 0.001 | 198 | MINAHASA SELATAN | 0.001 |
| 19 | TAPANULI SELATAN | 0.002 | 79 | TEBING TINGGI | 0.001 | 139 | SURAKARTA | 0.001 | 199 | MINAHASA UTARA | 0.001 |
| 20 | TAPANULI TENGAH | 0.002 | 80 | MEDAN | 0.001 | 140 | SALATIGA | 0.001 | 200 | MINAHASA TENGGARA | 0.001 |
| 21 | DAIRI | 0.002 | 81 | PADANG PARIAMAN | 0.001 | 141 | TEGAL | 0.001 | 201 | MANADO | 0.001 |
| 22 | PADANG LAWAS UTARA | 0.002 | 82 | SOLOK SELATAN | 0.001 | 142 | BANTUL | 0.001 | 202 | BITUNG | 0.001 |
| 23 | PADANG LAWAS | 0.002 | 83 | DHARMASRAYA | 0.001 | 143 | GUNUNG KIDUL | 0.001 | 203 | BANGGAI | 0.001 |
| 24 | PADANGSIDIMPUAN | 0.002 | 84 | PASAMAN BARAT | 0.001 | 144 | SLEMAN | 0.001 | 204 | PALU | 0.001 |
| 25 | PESISIR SELATAN | 0.002 | 85 | PAYAKUMBUH | 0.001 | 145 | YOGYAKARTA | 0.001 | 205 | GOWA | 0.001 |
| 26 | TANAH DATAR | 0.002 | 86 | PARIAMAN | 0.001 | 146 | PACITAN | 0.001 | 206 | MAROS | 0.001 |
| 27 | AGAM | 0.002 | 87 | KUANTAN SINGINGI | 0.001 | 147 | PONOROGO | 0.001 | 207 | PANGKAJENE DAN KEPULAUAN | 0.001 |
| 28 | SAWAH LUNTO | 0.002 | 88 | INDRAGIRI HULU | 0.001 | 148 | TRENGGALEK | 0.001 | 208 | BARRU | 0.001 |
| 29 | BUKITTINGGI | 0.002 | 89 | PELALAWAN | 0.001 | 149 | TULUNGAGUNG | 0.001 | 209 | SOPPENG | 0.001 |
| 30 | LAMPUNG TIMUR | 0.002 | 90 | SIAK | 0.001 | 150 | BLITAR | 0.001 | 210 | WAJO | 0.001 |
| 31 | KEPULAUAN ANAMBAS | 0.002 | 91 | KAMPAR | 0.001 | 151 | KEDIRI | 0.001 | 211 | SIDENRENG RAPPANG | 0.001 |
| 32 | SUMEDANG | 0.002 | 92 | ROKAN HULU | 0.001 | 152 | SIDOARJO | 0.001 | 212 | PINRANG | 0.001 |
| 33 | SUBANG | 0.002 | 93 | BENGKALIS | 0.001 | 153 | MOJOKERTO | 0.001 | 213 | TANA TORAJA | 0.001 |
| 34 | PURWAKARTA | 0.002 | 94 | ROKAN HILIR | 0.001 | 154 | JOMBANG | 0.001 | 214 | LUWU UTARA | 0.001 |
| 35 | SUKABUMI | 0.002 | 95 | DUMAI | 0.001 | 155 | MADIUN | 0.001 | 215 | LUWU TIMUR | 0.001 |
| 36 | TASIKMALAYA | 0.002 | 96 | KERINCI | 0.001 | 156 | MAGETAN | 0.001 | 216 | TORAJA UTARA | 0.001 |
| 37 | BANYUMAS | 0.002 | 97 | BATANG HARI | 0.001 | 157 | NGAWI | 0.001 | 217 | MAKASSAR | 0.001 |
| 38 | SEMARANG | 0.002 | 98 | TEBO | 0.001 | 158 | BOJONEGORO | 0.001 | 218 | KONAWE | 0.001 |
| 39 | MAGELANG | 0.002 | 99 | BUNGO | 0.001 | 159 | TUBAN | 0.001 | 219 | KONAWE SELATAN | 0.001 |
| 40 | MALANG | 0.002 | 100 | SUNGAI PENUH | 0.001 | 160 | LAMONGAN | 0.001 | 220 | KOLAKA UTARA | 0.001 |
| 41 | BANYUWANGI | 0.002 | 101 | OGAN KOMERING ULU | 0.001 | 161 | MALANG | 0.001 | 221 | BAUBAU | 0.001 |
| 42 | SERANG | 0.002 | 102 | MUARA ENIM | 0.001 | 162 | PROBOLINGGO | 0.001 | 222 | GORONTALO | 0.001 |
| 43 | TANAH LAUT | 0.002 | 103 | OGAN KOMERING ULU TIMUR | 0.001 | 163 | MOJOKERTO | 0.001 | 223 | MALUKU TENGAH | 0.001 |
| 44 | KATINGAN | 0.002 | 104 | PALEMBANG | 0.001 | 164 | MADIUN | 0.001 | 224 | BURU | 0.001 |
| 45 | TOMOHON | 0.002 | 105 | PRABUMULIH | 0.001 | 165 | SURABAYA | 0.001 | 225 | AMBON | 0.001 |
| 46 | KOTAMOBAGU | 0.002 | 106 | LUBUKLINGGAU | 0.001 | 166 | BATU | 0.001 | 226 | SORONG | 0.001 |
| 47 | MOROWALI | 0.002 | 107 | BENGKULU SELATAN | 0.001 | 167 | TANGERANG | 0.001 | 227 | MERAUKE | 0.001 |
| 48 | POSO | 0.002 | 108 | REJANG LEBONG | 0.001 | 168 | TANGERANG | 0.001 | 228 | MIMIKA | 0.001 |
| 49 | BANTAENG | 0.002 | 109 | LAMPUNG TENGAH | 0.001 | 169 | CILEGON | 0.001 |  |  |  |
| 50 | PALOPO | 0.002 | 110 | BANDAR LAMPUNG | 0.001 | 170 | JEMBRANA | 0.001 |  |  |  |
| 51 | KOLAKA | 0.002 | 111 | BANGKA | 0.001 | 171 | BANGLI | 0.001 |  |  |  |
| 52 | BONE BOLANGO | 0.002 | 112 | BELITUNG | 0.001 | 172 | LOMBOK BARAT | 0.001 |  |  |  |
| 53 | TERNATE | 0.002 | 113 | BANGKA BARAT | 0.001 | 173 | SUMBAWA BARAT | 0.001 |  |  |  |
| 54 | TIDORE KEPULAUAN | 0.002 | 114 | BANGKA TENGAH | 0.001 | 174 | MATARAM | 0.001 |  |  |  |
| 55 | ACEH SINGKIL | 0.001 | 115 | BELITUNG TIMUR | 0.001 | 175 | BIMA | 0.001 |  |  |  |
| 56 | ACEH TENGGARA | 0.001 | 116 | PANGKALPINANG | 0.001 | 176 | KOTAWARINGIN BARAT | 0.001 |  |  |  |
| 57 | ACEH BARAT | 0.001 | 117 | KARIMUN | 0.001 | 177 | BARITO SELATAN | 0.001 |  |  |  |
| 58 | PIDIE | 0.001 | 118 | BINTAN | 0.001 | 178 | BARITO UTARA | 0.001 |  |  |  |
| 59 | ACEH UTARA | 0.001 | 119 | NATUNA | 0.001 | 179 | LAMANDAU | 0.001 |  |  |  |
| 60 | ACEH TAMIANG | 0.001 | 120 | BATAM | 0.001 | 180 | GUNUNG MAS | 0.001 |  |  |  |

**Appendix 1D: Weights for current smokers among men**

| No | District | Weight | No | District | Weight | No | District | Weight |
| --- | --- | --- | --- | --- | --- | --- | --- | --- |
| 1 | PAGAR ALAM | 0.343 | 61 | BUKITTINGGI | 0.001 | 121 | BANDUNG | 0.001 |
| 2 | TEMANGGUNG | 0.174 | 62 | PAYAKUMBUH | 0.001 | 122 | CIREBON | 0.001 |
| 3 | BENER MERIAH | 0.093 | 63 | PARIAMAN | 0.001 | 123 | BEKASI | 0.001 |
| 4 | CIAMIS | 0.036 | 64 | KUANTAN SINGINGI | 0.001 | 124 | CIMAHI | 0.001 |
| 5 | BANTAENG | 0.016 | 65 | INDRAGIRI HULU | 0.001 | 125 | TASIKMALAYA | 0.001 |
| 6 | KARO | 0.007 | 66 | PELALAWAN | 0.001 | 126 | BANJAR | 0.001 |
| 7 | DAIRI | 0.002 | 67 | SIAK | 0.001 | 127 | CILACAP | 0.001 |
| 8 | ACEH SINGKIL | 0.001 | 68 | KAMPAR | 0.001 | 128 | BANYUMAS | 0.001 |
| 9 | ACEH TENGGARA | 0.001 | 69 | ROKAN HULU | 0.001 | 129 | PURBALINGGA | 0.001 |
| 10 | ACEH TENGAH | 0.001 | 70 | BENGKALIS | 0.001 | 130 | KEBUMEN | 0.001 |
| 11 | ACEH BARAT | 0.001 | 71 | ROKAN HILIR | 0.001 | 131 | PURWOREJO | 0.001 |
| 12 | ACEH BESAR | 0.001 | 72 | PEKANBARU | 0.001 | 132 | MAGELANG | 0.001 |
| 13 | PIDIE | 0.001 | 73 | DUMAI | 0.001 | 133 | BOYOLALI | 0.001 |
| 14 | BIREUEN | 0.001 | 74 | KERINCI | 0.001 | 134 | KLATEN | 0.001 |
| 15 | ACEH UTARA | 0.001 | 75 | MERANGIN | 0.001 | 135 | SUKOHARJO | 0.001 |
| 16 | ACEH TAMIANG | 0.001 | 76 | SAROLANGUN | 0.001 | 136 | WONOGIRI | 0.001 |
| 17 | NAGAN RAYA | 0.001 | 77 | BATANG HARI | 0.001 | 137 | KARANGANYAR | 0.001 |
| 18 | ACEH JAYA | 0.001 | 78 | MUARO JAMBI | 0.001 | 138 | SRAGEN | 0.001 |
| 19 | PIDIE JAYA | 0.001 | 79 | TEBO | 0.001 | 139 | GROBOGAN | 0.001 |
| 20 | BANDA ACEH | 0.001 | 80 | BUNGO | 0.001 | 140 | BLORA | 0.001 |
| 21 | SABANG | 0.001 | 81 | JAMBI | 0.001 | 141 | REMBANG | 0.001 |
| 22 | LANGSA | 0.001 | 82 | SUNGAI PENUH | 0.001 | 142 | PATI | 0.001 |
| 23 | LHOKSEUMAWE | 0.001 | 83 | OGAN KOMERING ULU | 0.001 | 143 | KUDUS | 0.001 |
| 24 | TAPANULI SELATAN | 0.001 | 84 | MUARA ENIM | 0.001 | 144 | JEPARA | 0.001 |
| 25 | TAPANULI TENGAH | 0.001 | 85 | OGAN KOMERING ULU TIMUR | 0.001 | 145 | DEMAK | 0.001 |
| 26 | TAPANULI UTARA | 0.001 | 86 | PALEMBANG | 0.001 | 146 | SEMARANG | 0.001 |
| 27 | TOBA | 0.001 | 87 | PRABUMULIH | 0.001 | 147 | KENDAL | 0.001 |
| 28 | LABUHAN BATU | 0.001 | 88 | LUBUKLINGGAU | 0.001 | 148 | BATANG | 0.001 |
| 29 | ASAHAN | 0.001 | 89 | BENGKULU SELATAN | 0.001 | 149 | PEKALONGAN | 0.001 |
| 30 | SIMALUNGUN | 0.001 | 90 | REJANG LEBONG | 0.001 | 150 | TEGAL | 0.001 |
| 31 | DELI SERDANG | 0.001 | 91 | BENGKULU UTARA | 0.001 | 151 | MAGELANG | 0.001 |
| 32 | LANGKAT | 0.001 | 92 | BENGKULU | 0.001 | 152 | SURAKARTA | 0.001 |
| 33 | HUMBANG HASUNDUTAN | 0.001 | 93 | LAMPUNG TIMUR | 0.001 | 153 | SALATIGA | 0.001 |
| 34 | SAMOSIR | 0.001 | 94 | LAMPUNG TENGAH | 0.001 | 154 | SEMARANG | 0.001 |
| 35 | SERDANG BEDAGAI | 0.001 | 95 | TULANGBAWANG | 0.001 | 155 | TEGAL | 0.001 |
| 36 | BATU BARA | 0.001 | 96 | PRINGSEWU | 0.001 | 156 | BANTUL | 0.001 |
| 37 | PADANG LAWAS UTARA | 0.001 | 97 | BANDAR LAMPUNG | 0.001 | 157 | GUNUNG KIDUL | 0.001 |
| 38 | PADANG LAWAS | 0.001 | 98 | METRO | 0.001 | 158 | SLEMAN | 0.001 |
| 39 | LABUHAN BATU SELATAN | 0.001 | 99 | BANGKA | 0.001 | 159 | YOGYAKARTA | 0.001 |
| 40 | LABUHAN BATU UTARA | 0.001 | 100 | BELITUNG | 0.001 | 160 | PACITAN | 0.001 |
| 41 | SIBOLGA | 0.001 | 101 | BANGKA BARAT | 0.001 | 161 | PONOROGO | 0.001 |
| 42 | TANJUNG BALAI | 0.001 | 102 | BANGKA TENGAH | 0.001 | 162 | TRENGGALEK | 0.001 |
| 43 | PEMATANG SIANTAR | 0.001 | 103 | BELITUNG TIMUR | 0.001 | 163 | TULUNGAGUNG | 0.001 |
| 44 | TEBING TINGGI | 0.001 | 104 | PANGKALPINANG | 0.001 | 164 | BLITAR | 0.001 |
| 45 | MEDAN | 0.001 | 105 | KARIMUN | 0.001 | 165 | KEDIRI | 0.001 |
| 46 | BINJAI | 0.001 | 106 | BINTAN | 0.001 | 166 | MALANG | 0.001 |
| 47 | PADANGSIDIMPUAN | 0.001 | 107 | NATUNA | 0.001 | 167 | BANYUWANGI | 0.001 |
| 48 | GUNUNGSITOLI | 0.001 | 108 | KEPULAUAN ANAMBAS | 0.001 | 168 | PASURUAN | 0.001 |
| 49 | PESISIR SELATAN | 0.001 | 109 | BATAM | 0.001 | 169 | SIDOARJO | 0.001 |
| 50 | SOLOK | 0.001 | 110 | TANJUNG PINANG | 0.001 | 170 | MOJOKERTO | 0.001 |
| 51 | TANAH DATAR | 0.001 | 111 | BOGOR | 0.001 | 171 | JOMBANG | 0.001 |
| 52 | PADANG PARIAMAN | 0.001 | 112 | BANDUNG | 0.001 | 172 | NGANJUK | 0.001 |
| 53 | AGAM | 0.001 | 113 | KUNINGAN | 0.001 | 173 | MADIUN | 0.001 |
| 54 | LIMA PULUH KOTA | 0.001 | 114 | CIREBON | 0.001 | 174 | MAGETAN | 0.001 |
| 55 | SOLOK SELATAN | 0.001 | 115 | SUMEDANG | 0.001 | 175 | NGAWI | 0.001 |
| 56 | DHARMASRAYA | 0.001 | 116 | SUBANG | 0.001 | 176 | BOJONEGORO | 0.001 |
| 57 | PASAMAN BARAT | 0.001 | 117 | PURWAKARTA | 0.001 | 177 | TUBAN | 0.001 |
| 58 | PADANG | 0.001 | 118 | KARAWANG | 0.001 | 178 | LAMONGAN | 0.001 |
| 59 | SOLOK | 0.001 | 119 | BEKASI | 0.001 | 179 | GRESIK | 0.001 |
| 60 | SAWAH LUNTO | 0.001 | 120 | SUKABUMI | 0.001 | 180 | KEDIRI | 0.001 |

| No | District | Weight | No | District | Weight |
| --- | --- | --- | --- | --- | --- |
| 181 | BLITAR | 0.001 | 241 | MINAHASA SELATAN | 0.001 |
| 182 | MALANG | 0.001 | 242 | MINAHASA UTARA | 0.001 |
| 183 | PROBOLINGGO | 0.001 | 243 | MINAHASA TENGGARA | 0.001 |
| 184 | PASURUAN | 0.001 | 244 | MANADO | 0.001 |
| 185 | MOJOKERTO | 0.001 | 245 | BITUNG | 0.001 |
| 186 | MADIUN | 0.001 | 246 | TOMOHON | 0.001 |
| 187 | SURABAYA | 0.001 | 247 | KOTAMOBAGU | 0.001 |
| 188 | BATU | 0.001 | 248 | BANGGAI | 0.001 |
| 189 | TANGERANG | 0.001 | 249 | MOROWALI | 0.001 |
| 190 | TANGERANG | 0.001 | 250 | POSO | 0.001 |
| 191 | CILEGON | 0.001 | 251 | PALU | 0.001 |
| 192 | SERANG | 0.001 | 252 | BULUKUMBA | 0.001 |
| 193 | TANGERANG SELATAN | 0.001 | 253 | GOWA | 0.001 |
| 194 | JEMBRANA | 0.001 | 254 | MAROS | 0.001 |
| 195 | TABANAN | 0.001 | 255 | PANGKAJENE DAN KEPULAUAN | 0.001 |
| 196 | BADUNG | 0.001 | 256 | BARRU | 0.001 |
| 197 | BANGLI | 0.001 | 257 | SOPPENG | 0.001 |
| 198 | BULELENG | 0.001 | 258 | WAJO | 0.001 |
| 199 | DENPASAR | 0.001 | 259 | SIDENRENG RAPPANG | 0.001 |
| 200 | LOMBOK BARAT | 0.001 | 260 | PINRANG | 0.001 |
| 201 | SUMBAWA BARAT | 0.001 | 261 | ENREKANG | 0.001 |
| 202 | MATARAM | 0.001 | 262 | LUWU | 0.001 |
| 203 | BIMA | 0.001 | 263 | TANA TORAJA | 0.001 |
| 204 | PONTIANAK | 0.001 | 264 | LUWU UTARA | 0.001 |
| 205 | SINGKAWANG | 0.001 | 265 | LUWU TIMUR | 0.001 |
| 206 | KOTAWARINGIN BARAT | 0.001 | 266 | TORAJA UTARA | 0.001 |
| 207 | KOTAWARINGIN TIMUR | 0.001 | 267 | MAKASSAR | 0.001 |
| 208 | KAPUAS | 0.001 | 268 | PAREPARE | 0.001 |
| 209 | BARITO SELATAN | 0.001 | 269 | PALOPO | 0.001 |
| 210 | BARITO UTARA | 0.001 | 270 | MUNA | 0.001 |
| 211 | LAMANDAU | 0.001 | 271 | KONAWE | 0.001 |
| 212 | GUNUNG MAS | 0.001 | 272 | KOLAKA | 0.001 |
| 213 | BARITO TIMUR | 0.001 | 273 | KONAWE SELATAN | 0.001 |
| 214 | TANAH LAUT | 0.001 | 274 | WAKATOBI | 0.001 |
| 215 | KATINGAN | 0.001 | 275 | KOLAKA UTARA | 0.001 |
| 216 | KOTABARU | 0.001 | 276 | KONAWE UTARA | 0.001 |
| 217 | BANJAR | 0.001 | 277 | KENDARI | 0.001 |
| 218 | TAPIN | 0.001 | 278 | BAUBAU | 0.001 |
| 219 | HULU SUNGAI SELATAN | 0.001 | 279 | BONE BOLANGO | 0.001 |
| 220 | HULU SUNGAI TENGAH | 0.001 | 280 | GORONTALO | 0.001 |
| 221 | TABALONG | 0.001 | 281 | MALUKU TENGAH | 0.001 |
| 222 | TANAH BUMBU | 0.001 | 282 | BURU | 0.001 |
| 223 | BALANGAN | 0.001 | 283 | AMBON | 0.001 |
| 224 | BANJARMASIN | 0.001 | 284 | TERNATE | 0.001 |
| 225 | BANJAR BARU | 0.001 | 285 | TIDORE KEPULAUAN | 0.001 |
| 226 | PASER | 0.001 | 286 | MANOKWARI | 0.001 |
| 227 | KUTAI BARAT | 0.001 | 287 | SORONG | 0.001 |
| 228 | KUTAI KARTANEGARA | 0.001 | 288 | MERAUKE | 0.001 |
| 229 | KUTAI TIMUR | 0.001 | 289 | JAYAPURA | 0.001 |
| 230 | BERAU | 0.001 | 290 | NABIRE | 0.001 |
| 231 | PENAJAM PASER UTARA | 0.001 | 291 | MIMIKA | 0.001 |
| 232 | BALIKPAPAN | 0.001 | 292 | JAYAPURA | 0.001 |
| 233 | SAMARINDA | 0.001 |  |  |  |
| 234 | BONTANG | 0.001 |  |  |  |
| 235 | MALINAU | 0.001 |  |  |  |
| 236 | BULUNGAN | 0.001 |  |  |  |
| 237 | TARAKAN | 0.001 |  |  |  |
| 238 | MINAHASA | 0.001 |  |  |  |
| 239 | KEPULAUAN SANGIHE | 0.001 |  |  |  |
| 240 | KEPULAUAN TALAUD | 0.001 |  |  |  |

**Appendix 2: Robustness check of impact using all districts**

| **Outcome** | **Year** | **Treated (%)** | **Synthetic (%)** | **Absolute Diff (%)** | **Relative Diff (%)** |
| --- | --- | --- | --- | --- | --- |
|  | [1] | [2] | [3] | [4]=[2]-[3] | [5]=[4]/[3] |
|  |  |  |  |  |  |
| Ever Smoker: Boys | 2013 | 30.0 | 30.1 | -0.1 | 0.02 |
|  | 2018 | 21.7 | 29.5 | -7.9 | -27.2 |
|  | 2023 | 23.4 | 36.5 | -13.1 | -35.1 |
|  |  |  |  |  |  |
| Current Smoker: Boys | 2013 | 24.8 | 24.7 | 0.0 | 0.02 |
|  | 2018 | 13.1 | 21.2 | -8.1 | -27.2 |
|  | 2023 | 19.2 | 34.0 | -14.8 | -35.1 |
|  |  |  |  |  |  |
| Ever Smoker: Adult Men | 2013 | 88.3 | 91.4 | -3.1 | 0.02 |
|  | 2018 | 82.0 | 84.3 | -2.3 | -27.2 |
|  | 2023 | 79.8 | 85.4 | -5.6 | -35.1 |
|  |  |  |  |  |  |
| Current Smoker: Adult Men | 2013 | 78.6 | 79.9 | -1.2 | 0.02 |
|  | 2018 | 71.3 | 73.2 | -2.0 | -27.2 |
|  | 2023 | 69.0 | 63.4 | 5.6 | -35.1 |

Note: Treatment effects in column [4] are calculated by subtracting the synthetic control’s smoking prevalence from the observed prevalence in the treated unit (Kota Bogor). Column [5] expresses this difference as a percentage of the synthetic control value. The synthetic control values were derived by applying donor district weights (see Table 2) to the smoking prevalence rates of contributing districts in the donor pool.
